# Natural Compounds Tapinarof and *Galactomyces* Ferment Filtrate Downregulate IL-33 Expression *via* the AHR/IL-37 Axis in Human Keratinocytes

**DOI:** 10.3389/fimmu.2022.745997

**Published:** 2022-05-19

**Authors:** Gaku Tsuji, Akiko Hashimoto-Hachiya, Tomoyo Matsuda-Taniguchi, Ayako Takai-Yumine, Masaki Takemura, Xianghong Yan, Masutaka Furue, Takeshi Nakahara

**Affiliations:** ^1^Research and Clinical Center for Yusho and Dioxin, Kyushu University, Fukuoka, Japan; ^2^Department of Dermatology, Graduate School of Medical Sciences, Kyushu University, Fukuoka, Japan; ^3^Science Communications, Procter & Gamble (P&G) Innovation Godo Kaisha, Kobe, Japan

**Keywords:** IL-33, AHR (aryl hydrocarbon receptor), IL-37, atopic dermatitis, psoriasis

## Abstract

Interleukin (IL)-37 suppresses systemic and local inflammation. It is expressed in the epidermis, the external layer of the skin, and is decreased in inflammatory skin diseases including atopic dermatitis (AD) and psoriasis. Therefore, an agent applied topically on the skin that can increase IL-37 could be promising for treating AD and psoriasis; however, the mechanism regulating IL-37 remains largely unknown. Given that IL-37 expression is induced in differentiated keratinocytes, a major component of the epidermis, and that activation of aryl hydrocarbon receptor (AHR), a ligand-activated transcription factor, promotes keratinocyte differentiation, we hypothesized that AHR might be involved in the IL-37 expression in human keratinocytes. We analyzed normal epidermal human keratinocytes (NHEKs) treated with tapinarof and *Galactomyces* ferment filtrate (GFF), which are potent AHR modulators. We found that tapinarof and GFF upregulated IL-37 in NHEKs, which was canceled by the knockdown of AHR using siRNA transfection, indicating that AHR mediates IL-37 expression in NHEKs. Furthermore, we found that the knockdown of IL-37 resulted in the upregulation of IL-33, an alarmin cytokine with crucial roles in the pathogenesis of AD and psoriasis. These findings suggest that IL-37 negatively regulates IL-33 expression in NHEKs. Finally, we examined whether tapinarof and GFF treatment modulates IL-33 expression in NHEKs. Such treatment inhibited IL-33 expression, which was partially reversed by the knockdown of either AHR or IL-37. Taken together, our findings provide the first evidence that tapinarof and GFF could have potential to prevent IL-33-overexpressing disorders such as AD and psoriasis *via* the AHR/IL-37 axis.

## Introduction

Interleukin (IL)-37, an anti-inflammatory cytokine of the IL-1 family, has been reported to suppress the production of inflammatory mediators in the immune response (1). IL-37 suppresses Toll-like receptor-, IL-1-, IL-18-, IL-33-, and IL-36-mediated inflammation (2). The mechanisms behind this include suppression of the MyD88-mediated signaling pathway (3), nuclear factor-κB (4), mitogen activation protein kinase (5), and mTOR phosphorylation (6). Based on these findings, IL-37 is expected to have potential to attenuate the development of various inflammatory diseases including inflammatory skin diseases such as atopic dermatitis (AD) and psoriasis through its anti-inflammatory effects.

AD is an eczematous skin disease commonly encountered in daily practice. Its clinical features include skin inflammation, impaired skin barrier function, and chronic pruritus. The pathogenesis of AD is still unclear, but type 2 cytokines, such as IL-4 and IL-13, are thought to be the main factors involved (7). In a mouse model of atopic dermatitis induced by the topical application of MC903, a vitamin D analog, it was reported that the development of atopic dermatitis was suppressed by the knock-in of human IL-37 (8). In humans, it has also been reported that skin lesional IL-37 levels are decreased in AD compared with those in healthy subjects (9). These findings suggest that IL-37 has a protective role against the development of AD.

Psoriasis is a common chronic inflammatory disease of the skin affecting 0.6%–3% of the global population. The skin lesions manifest as multiple scaly erythema on the face, scalp, trunk, and upper and lower extremities, which places a significant physical and psychological burden on affected patients, resulting in a decreased quality of life (10). The therapeutic efficacy of anti-TNF-α/IL-23/IL-17A biologics for psoriasis suggests that the TNF-α/IL-23/IL-17A axis plays a central role in its pathogenesis (11). Although the precise role of IL-37 in the development of psoriasis remains unclear, intradermal injection of recombinant human IL-37 into a murine imiquimod-induced psoriasis model reportedly demonstrated a trend toward a protective effect (12). It has also been reported that IL-37 expression is markedly downregulated in biopsies from lesional psoriasis human skin compared with the level in paired samples of non-lesional skin (12, 13). These findings suggest that IL-37 may have potential to prevent the development of psoriasis, in addition to AD.

The above background suggests the potential for a new therapeutic strategy targeting IL-37 for AD and psoriasis, but the regulatory mechanism of IL-37 in the skin remains unclear. In this study, we found that the expression of IL-37 in human keratinocytes, a major component of the skin, is mediated by aryl hydrocarbon receptor (AHR), a ligand-activated transcription factor. AHR is activated by various ligands including dioxins, PAHs, azole drugs, and plant-derived extracts, and is characterized by different biological responses depending on the ligand (14). Currently, agents that act on the AHR for therapeutic purposes (named therapeutic AHR-modulating agents: TAMAs) are being developed for the treatment of AD and psoriasis clinically (15). Among them, tapinarof is a natural antioxidative compound extracted from *Cordyceps sinensis*. Its topical application has been evaluated in Phase II trials for AD (16) and psoriasis (17), demonstrating therapeutic efficacy. We previously reported that tapinarof improved the decreases of filaggrin and loricrin in AD *via* AHR, which is one of the mechanisms behind its therapeutic effects on AD (18). We also previously reported *Galactomyces* ferment filtrate (GFF), a type of antioxidative fermented yeast product, as a moisturizing cosmetic product, as having the same mechanism of action as tapinarof. GFF also prevents the Th2 cytokine-mediated reduction of skin barrier proteins such as filaggrin and loricrin in an AHR-dependent fashion (19). These findings prompted us to propose a beneficial role of antioxidative AHR agonists in skin barrier differentiation (20).

In the skin, IL-37 protein is reportedly co-expressed with loricrin and strongly expressed in the granular layer of the epidermis (21). It is thus possible that IL-37 expression is coordinately regulated by keratinocyte differentiation. Considering that the actions of tapinarof and GFF on AHR drive keratinocyte differentiation, we hypothesized that tapinarof and GFF treatment might modulate IL-37 expression in human keratinocytes. We found that the AHR-mediated IL-37 upregulation attenuates IL-33 expression which is one of the critical cytokines responsible for AD and psoriasis.

## Materials and Methods

### Reagents and Antibodies

Tapinarof (MedChemExpress, Monmouth Junction, NJ, USA) was dissolved in dimethyl sulfoxide (DMSO; Nacalai Tesque, Kyoto, Japan) and stored at −80°C until used in the experiments. GFF was obtained from P&G Innovation Godo Kaisha (Kobe, Japan). Anti-human IL-37 polyclonal goat antibody (R&D Systems, Minneapolis, MN, USA), anti-human IL-33 monoclonal mouse antibody, anti-human IL-36γ/IL-1F9 monoclonal mouse antibody (Abcam, Cambridge, UK), anti-phosphorylated ERK-1/2 rabbit monoclonal antibody (Thr202/Tyr204), anti-ERK-1/2 rabbit monoclonal antibody, anti-phosphorylated p38 rabbit monoclonal antibody (Thr180/Tyr182), anti-p38 rabbit monoclonal antibody, anti-phosphorylated JNK rabbit monoclonal antibody (Thr183/Tyr185), anti-JNK rabbit polyclonal antibody, anti-AHR monoclonal rabbit antibody, and anti-human β-actin monoclonal mouse antibody (Cell Signaling Technology, Danvers, MA, USA) were used for western blotting. Anti-human IL-37 polyclonal rabbit antibody and IgG rabbit polyclonal antibody (Abcam) were used for immunofluorescence.

### Cell Culture

Normal human epidermal keratinocytes (NHEKs) purchased from Lonza (Basel, Switzerland) were grown in serum-free keratinocyte culture medium, namely, KBM Gold Basal Medium (Lonza) supplemented with bovine pituitary extract, recombinant epidermal growth factor, insulin, hydrocortisone, transferrin, and epinephrine, at 37°C in 5% CO_2_. The growth medium was replenished every 2–3 days. Cells reaching confluence (70%–90%) were disaggregated with 0.25 mg/mL trypsin/0.01% ethylenediaminetetraacetic acid and then sub-cultured. NHEKs at the second to fourth passages were utilized for the experiments. For 3D cultured NHEKs, a human epidermis model (Raft 3D cell culture kit; Lonza) derived from newborn foreskin was used. Neonatal normal human dermal fibroblasts (Lonza) and neonatal normal human epidermal keratinocytes (Lonza) placed as a monolayer were stratified to full thickness in accordance with the manufacturer’s instructions, in a humidified atmosphere with 5% CO_2_ at 37°C. On the 10th day, either tapinarof or GFF was added to the lower liquid phase of the 3D cell tissue.

### Cell Viability Analysis

The effects of tapinarof and GFF on NHEK viability were measured by a water-soluble tetrazolium salt (WST-1) assay. We utilized Premix WST-1 Cell Proliferation Assay System (Takara Bio, Shiga, Japan). NHEKs were seeded at 2×10^4^ cells/well in 96-well microplates and incubated for 24 h. To examine the toxicity of tapinarof and GFF, the cells were treated with the indicated concentrations of either tapinarof or GFF for 24 h. WST-1 solution was then added to the cells for 4 h. The absorbance of each sample was measured using a microplate reader (DTX 800 Multimode Detector; Beckman Coulter, Brea, CA, USA) with filters at 450 nm and a reference wavelength at 620 nm. The results are presented as the relative absorbance compared with untreated NHEKs. No decrease in viability was observed at the concentrations used in this experiment, which is consistent with our previous studies (18, 19). The results are shown in **Supplementary Figure S1**.

### Transfection of siRNAs Against AHR and IL-37

Small interfering RNAs (siRNAs) against AHR (AHR siRNA, s1200), IL-37 (IL-37 siRNA, s25954), and non-targeting siRNA (control siRNA) were obtained from Ambion (Austin, TX, USA). Cells were incubated in the culture medium with a mixture containing 5 nM siRNA and HiPerFect Transfection Reagent (Qiagen, Venlo, The Netherlands) for 48 h and then used for further experiments. The knockdown efficiencies of siRNA transfection are presented in **Supplementary Figure S2**.

### Quantitative Reverse-Transcription (qRT)-PCR

Total RNA was extracted using an RNeasy Mini Kit (Qiagen), followed by the production of cDNA by reverse transcription using the PrimeScript RT reagent kit (Takara Bio). qRT-PCR was performed on a CFX Connect Real-time PCR Detection System (Bio-Rad, Hercules, CA, USA). Gene expression levels of IL-37 were determined by TaqMan qRT-PCR. We utilized TaqMan Fast Advanced Master Mix (Thermo Fisher Scientific, Waltham, MA, USA). Amplification was started as the first step at 50°C for 2 min and 95°C for 20 s, followed by 45 cycles of qRT-PCR at 95°C for 3 s and at 60°C for 10 s as the second step. mRNA expression was measured in triplicate with normalization using the housekeeping gene YWHAZ. Gene expression levels of IL-33, IL-36γ, and AHR were determined by SYBR green-based qRT-PCR utilizing TB Green Premix Ex Taq (Takara Bio). Amplification was started as the first step at 95°C for 30 s, followed by 40 cycles of 95°C for 5 s and 60°C for 20 s as the second step. mRNA expression was measured in triplicate with normalization using the housekeeping gene β-actin. Primer sequences are shown in **Supplementary Table 1**.

### Western Blotting Analysis

NHEKs were incubated for 5 min in Complete Lysis-M (Roche Diagnostics, Rotkreuz, Switzerland) for western blotting analysis of IL-37, IL-33, IL-36γ, or AHR. The protein concentration of collected cell lysates was measured using a BCA protein assay kit (Thermo Fisher Scientific, Rockford, IL, USA), following the manufacturer’s protocol. Equal amounts of protein were mixed with 10% NuPage sample reducing agent 10× (Thermo Fisher Scientific) and 25% NuPage LDS sample buffer 4× (Thermo Fisher Scientific), and then heated at 70°C for 10 min. Next, the proteins were loaded and run on 4%–12% Bis-Tris Gel (Thermo Fisher Scientific) at 200 V for 20 min and transferred to a PVDF membrane (Merck Millipore, Burlington, MA, USA). The membranes were blocked in WesternBreeze blocker/diluent (Thermo Fisher Scientific) for 30 min and then probed with primary antibody overnight at 4°C. They were subsequently incubated with anti-mouse horseradish peroxidase-conjugated IgG secondary antibody (Cell Signaling Technology) or anti-goat horseradish peroxidase-conjugated IgG secondary antibody (Promega Corporation, Madison, WI, USA). Protein bands were visualized with Super Signal West Pico Plus Chemiluminescent Substrate (Thermo Fisher Scientific) using the ChemiDoc Touch Imaging System (Bio-Rad).

### Immunofluorescence and Confocal Laser Scanning Microscopy

NHEKs were cultured on slides (Lab-Tek, Rochester, NY, USA). These slides were then washed in phosphate-buffered saline (PBS), fixed with acetone for 10 min, and blocked using 10% bovine serum albumin (Roche Diagnostics, Basel, Switzerland) in PBS for 30 min for the staining of IL-37. Samples were incubated with primary anti-human IL-37 polyclonal rabbit antibody (1:100) (Abcam) or IgG rabbit polyclonal antibody (Abcam) in Western Breeze Blocker/Diluent (Thermo Fisher Scientific) overnight at 4°C. The slides were then washed with PBS before incubation with anti-rabbit secondary antibody (Alexa Fluor 546; Molecular Probes, Eugene, OR, USA) for 1 h at room temperature. After nuclear staining with 4′,6-diamidino-2-phenylindole (DAPI), the slides were mounted with UltraCruz mounting medium (Santa Cruz Biotechnology, Dallas, TX, USA). All samples were analyzed using a D-Eclipse confocal laser scanning microscope (Nikon, Tokyo, Japan).

### DNA Microarray Analysis

Five hundred nanograms of pooled RNA isolated from NHEKs transfected with either control siRNA or IL-37 siRNA was used for microarray analysis with Clariom™ S Array, human (Applied Biosystem, Waltham, MA, USA). RNA samples derived from the NHEKs were reverse-transcribed to single-stranded cDNA using Genechip™ WT Amplification Kit (Affymetrix). The synthesis of double-stranded cDNA and cRNA was performed in accordance with the manufacturer’s instructions. The cRNA samples were hybridized onto the array chip at 45°C for 16 h and stained with streptavidin-phycoerythrin using GeneChip™ Fluidics Station 450 (Applied Biosystem). The imaging data were obtained with GeneChip™ Scanner 3000 (Affymetrix). The data were converted to CEL file format using Affymetrix GeneChip^®^ Command Console. The data were normalized with Transcriptome Analysis Console (Applied Biosystems). The cut-off was set as |fold change| ≥ 2 and the gene sets satisfying this cut-off were further subjected to software analysis (Multiple Experimental Viewer: https://sourceforge.net/projects/mev-tm4/) for the volcano plot and heat map analysis.

### Mouse Experiments Using Intradermal Injection of Human IL-37

BALB/c female mice were housed in a clean facility until 6–7 weeks of age by Japan SLC, Inc. (Shizuoka, Japan). The animal experiments were conducted in accordance with a protocol reviewed and approved by the Animal Facility Center of Kyushu University (A21-445-0, 2021–2023). The back skin of each mouse was injected intradermally for 6 or 24 h with 100 ng of human IL-37/IL-1F7b, CF (aa46-218; R&D Systems) dissolved with 80 µL of PBS. PBS solution was used as a control. Then, the back skin was incubated for 20 min at 37°C on 3.8% ammonium thiocyanate (Sigma-Aldrich, St. Louis, MI, USA) to collect the epidermal sheet. This sheet immersed in TRIZOL (Thermo Fischer Scientific) was homogenized by FastPrep homogenizer (MP Biomedicals, LLC, Irvine, CA, USA). Then, mRNA was purified using RNeasy Mini Kit (Qiagen).

### Statistical Analysis

Quantitative data are presented as mean ± standard error of the mean (S.E.M.). The statistical significance of differences between two groups was examined by Student’s unpaired two-tailed t-test or one-way ANOVA test within multiple groups, followed by Tukey’s *post hoc* test with a p-value of less than 0.05 being considered significant.

## Results

### Tapinarof and GFF Treatment Upregulated IL-37 *via* AHR in NHEKs

**Figure 1:**
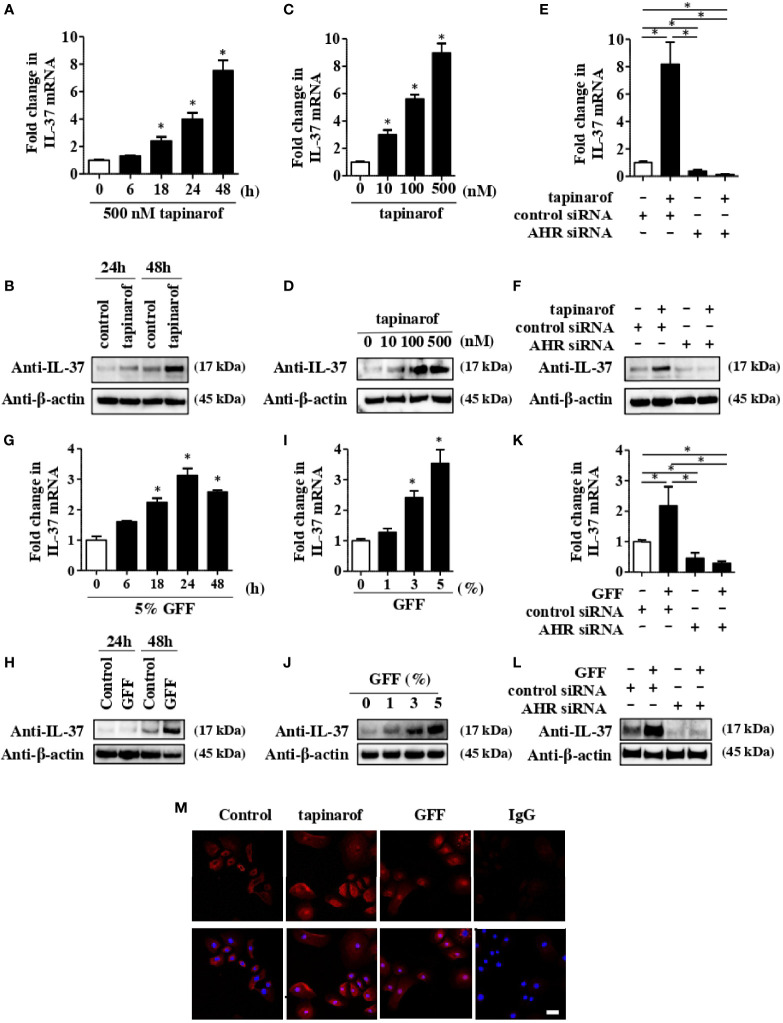
Tapinarof and GFF treatment upregulated IL-37 *via* AHR in NHEKs. Normal human epidermal keratinocytes (NHEKs) were treated with either tapinarof (500 nM) **(A, B)** or GFF **(G, H)** for the indicated period. NHEKs were treated with either tapinarof **(C, D)** or GFF **(I, J)** at the indicated dose for 48 h **(A, C, G, I)** The level of IL-37 mRNA was analyzed by qRT-PCR. Data are expressed as mean ± S.E.M.; n = 3 for each group. Statistically significant differences in the expression of control and tapinarof- or GFF-treated NHEKs are presented: *p < 0.05. **(B, D, H, J)** Total cell lysates were prepared and subjected to western blotting analysis with an anti-IL-37 antibody. The data are representative of experiments repeated three times with similar results. **(E, F, K, L)** NHEKs were transfected with either control siRNA (control siRNA) or siRNA against AHR (AHR siRNA) and subsequently treated with either tapinarof (500 nM) or GFF (5%) for 48 h **(E, K)** The mRNA level of IL-37 was analyzed by qRT-PCR. Data are expressed as mean ± S.E.M.; n = 3 for each group. Statistically significant differences between the expression of control and tapinarof- or GFF-treated NHEKs are presented: *p < 0.05. **(F, L)** The level of protein of IL-37 was analyzed by western blotting with an anti-IL-37 antibody. The data are representative of experiments repeated three times with similar results. **(M)** Immunofluorescent staining analysis using confocal laser scanning microscopy. NHEKs under control treatment or treated with tapinarof (500 nM) or GFF (5%) for 12 h were stained with an anti-IL-37 antibody (primary antibody) and an Alexa Fluor 546-conjugated anti-rabbit IgG antibody (red: secondary antibody). DAPI (4′,6-diamidino-2-phenylindole) was utilized for nuclear staining. IgG: isotype negative control: The scale bar represents 25 μm. The data are representative of experiments repeated three times with similar results.

It has been reported that IL-37 is expressed in keratinocytes maintained in subconfluent monolayer culture and in skin equivalents (22). We first evaluated the level of IL-37 in NHEKs treated with tapinarof. qRT-PCR and western blotting analysis showed that tapinarof treatment (500 nM) increased the mRNA and protein levels of IL-37 in a time-dependent manner (**Figures 1A, B**). In addition, tapinarof treatment (0, 100, and 500 nM) for 48 h increased the mRNA and protein levels of IL-37 in a dose-dependent manner (**Figures 1C, D**). To further examine the involvement of AHR in the upregulation of IL-37 induced by tapinarof treatment, we transfected NHEKs with siRNA against AHR, which successfully knocked down the levels of mRNA and protein of AHR (**Supplementary Figure 2**), and then applied treatment with tapinarof (500 nM) for 48 h. qRT-PCR and western blotting analysis showed that knockdown of AHR canceled the tapinarof-induced upregulation of IL-37 mRNA and protein in NHEKs (**Figures 1E, F**). To further substantiate the mechanism by which AHR modulates IL-37 expression in NHEKs, we utilized GFF, another AHR modulator. We confirmed that 5% GFF treatment increased the mRNA and protein levels of IL-37 in a time-dependent manner (**Figures 1G, H**). In addition, GFF treatment for 48 h upregulated IL-37 at the mRNA and protein levels in a dose-dependent manner (**Figure 1I, J**). Mirroring the trend shown for tapinarof, knockdown of AHR abolished the GFF-induced upregulation of IL-37 mRNA (**Figure 1K**) and protein (**Figure 1L**).

To further determine the cellular localization of IL-37 in NHEKs, we performed immunofluorescent analysis using anti-IL-37 antibody on NHEKs treated with either tapinarof (500 nM) or 5% GFF for 24 h. We observed both cellular and nuclear IL-37 expression in NHEKs treated with either tapinarof or GFF (**Figure 1M**). In accordance with a previous study using human keratinocytes (23), using ELISA we could not detect secreted IL-37 in the culture supernatant of NHEKs treated with either tapinarof or GFF (data not shown).

### IL-37 Negatively Regulated IL-33 in NHEKs

**Figure 2:**
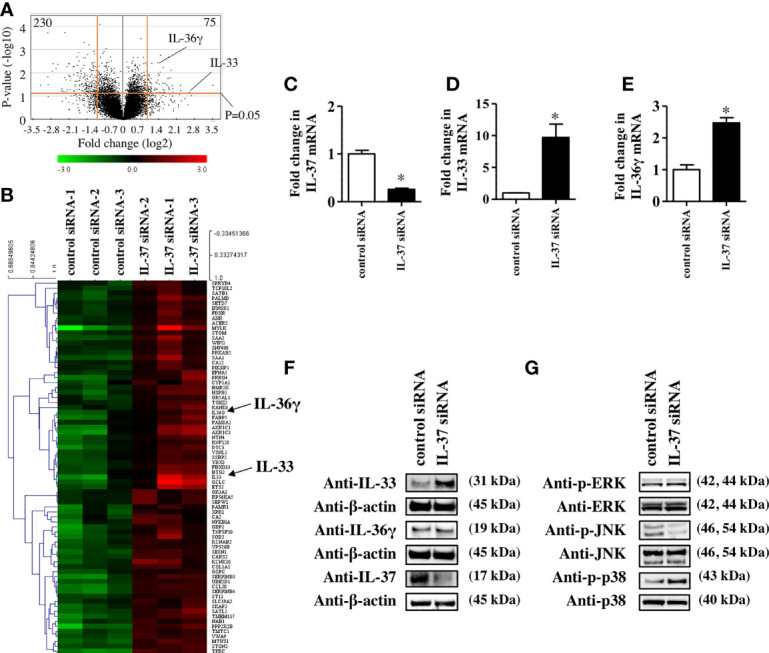
IL-37 negatively regulated IL-33 in NHEKs. **(A–G)** NHEKs were transfected with either control siRNA (control siRNA) or siRNA against IL-37 (IL-37 siRNA). **(A, B)** Microarray analysis. **(A)** Volcano plot for control siRNA- and IL-37 siRNA-treated NHEKs. Average log_2_(fold change) versus log_10_(p-value) for all genes is indicated. **(B)** Heat map of genes in NHEKs transfected with either control siRNA (left) or IL-37 siRNA. **(C–E)** qRT-PCR analysis. mRNA levels of IL-37 **(C)**, IL-33 **(D)**, and IL-36γ **(E)**. **(C–E)** Data are expressed as mean ± S.E.M.; n = 3 for each group. Statistically significant differences between the expression of control siRNA- and IL-37 siRNA-transfected NHEKs are presented: *p < 0.05. **(F)** Western blotting analysis with anti-IL-33, -IL-36γ, or -IL-37 antibody. **(G)** Western blotting analysis on the phosphorylation of MAPKs. **(F, G)** The data are representative of experiments repeated three times with similar results.

To further reveal the role of IL-37 in the skin, we examined how IL-37 knockdown altered gene expression in NHEKs. Microarray analysis showed that IL-37 knockdown significantly upregulated 121 genes and downregulated 329 genes (p<0.05) compared with the levels in control siRNA-transfected NHEKs (**Figure 2A**). The top 75 upregulated and 100 downregulated genes are listed in **Supplementary Tables 2****,** **3**. Although the IL-37 gene was not listed among the significantly downregulated genes in NHEKs with IL-37 knockdown, we confirmed that siRNA transfection against IL-37 successfully downregulated it using qRT-PCR analysis (**Figure 2C**). This may have been due to the design of the primers for IL-37 in the microarray. Among upregulated genes, we found that the mRNA levels of IL-33 and IL-36, members of the IL-1 family, were upregulated in NHEKs with IL-37 knockdown (**Figure 2B**). qRT-PCR analysis confirmed that IL-37 knockdown increased the levels of IL-33 and IL-36 mRNA (**Figures 2D, E**); however, western blotting analysis showed that IL-37 knockdown increased the protein level of IL-33 but not that of IL-36 in NHEKs (**Figure 2F**). Furthermore, western blotting analysis showed that IL-37 knockdown induced the phosphorylation of p38 of MAPKs (**Figure 2G**), which is required for IL-33 expression in NHEKs (24). Although it is desirable to further examine whether IL-37 negatively regulates IL-33 *in vivo*, since no homologous gene has been found in the mouse genome (1), *in vivo* experiments regarding IL-37 are challenging. Therefore, we examined whether human IL-37 could suppress the expression of murine Il-33 in the epidermis by the injection of human IL-37 into mice. We injected either PBS or human IL-37 (100 ng) into Balb/c mice intradermally. We extracted mRNAs from the epidermal sheet at 6 or 24 h after injection and then examined the mRNA levels of Il-33. qRT-PCR analysis showed that human IL-37 injection significantly reduced the mRNA levels of Il-33 (**Supplementary Figure S3**), indicating the possibility that IL-37 regulates IL-33 expression negatively in the epidermis *in vivo*.

### Tapinarof and GFF Treatment Downregulated IL-33 in NHEKs

**Figure 3:**
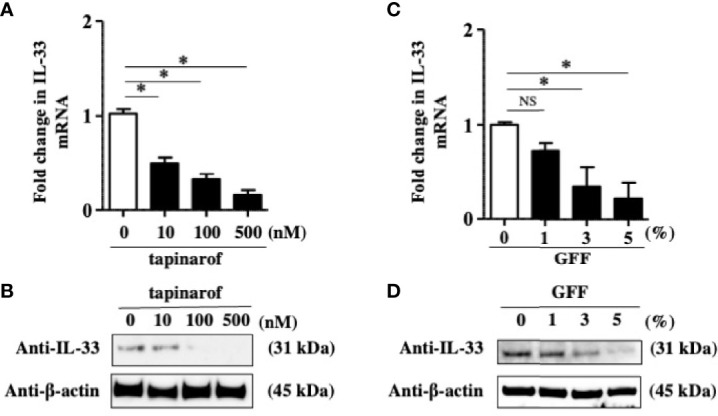
Tapinarof and GFF treatment downregulated IL-33 in NHEKs. **(A–D)** NHEKs were treated with either tapinarof (500 nM) or GFF (5%) for 48 h. **(A, C)** IL-33 mRNA expression was analyzed by qRT-PCR. Data are expressed as mean ± S.E.M.; n = 4 for each group. NS, not significant, *p < 0.05 (one-way ANOVA with Tukey’s multiple comparisons test). **(B, D)** IL-33 protein expression was analyzed by western blotting with an anti-IL-33 antibody. The data are representative of experiments repeated three times with similar results.

The data showing that a decrease of IL-37 is likely to result in the upregulation of IL-33 in NHEKs led us to think that tapinarof and GFF might inhibit IL-33 expression in NHEKs *via* the upregulation of IL-37. To clarify this, we treated NHEKs with either tapinarof (10, 100, and 500 nM) or GFF (1%, 3%, and 5%) for 48 h. qRT-PCR and western blotting analysis of IL-33 showed that tapinarof and GFF decreased the mRNA and protein levels of IL-33 in a dose-dependent manner in NHEKs (**Figures 3A–D**). This is consistent with our previous study showing that AHR activation downregulated IL-33 expression in keratinocytes (24). We further examined whether tapinarof and GFF can exert the same effect in 3D cultured NHEKs. We treated 3D cultured NHEKs with either tapinarof (500 nM) or GFF (5%) for 48 h. Western blotting analysis showed that tapinarof and GFF increased the protein levels of IL-37 while decreasing those of IL-33 in 3D cultured NHEKs (**Supplementary Figure S4**).

### Inhibitory Effect of Tapinarof and GFF Treatment on IL-33 Expression Was Partially Dependent on the AHR/IL-37 Axis in NHEKs

**Figure 4:**
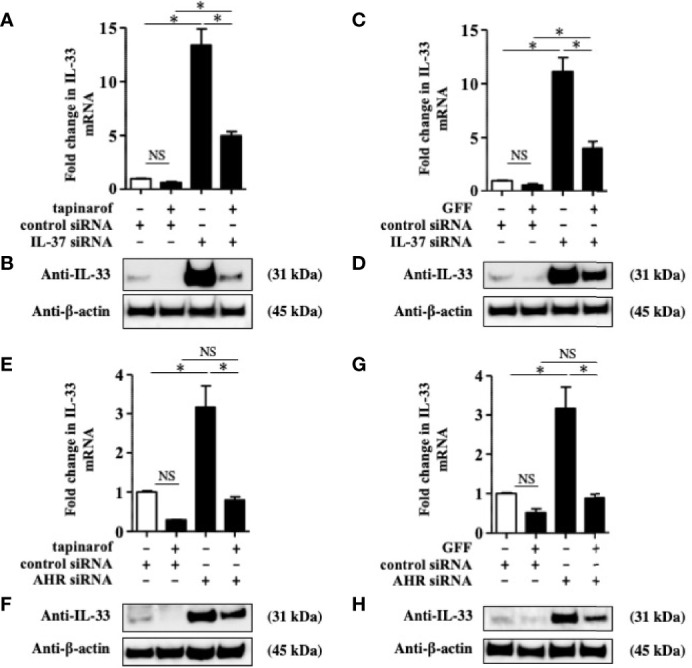
Inhibitory effect of tapinarof and GFF treatment on IL-33 expression was partially dependent on the AHR/IL-37 axis in NHEKs. **(A–H)** NHEKs transfected with control siRNA, siRNA against IL-37 (IL-37 siRNA), or AHR siRNA were treated with either tapinarof (500 nM) or GFF (5%) for 48 h. **(A, C, E, G)** IL-33 expression was analyzed by qRT-PCR. Data are expressed as mean ± S.E.M.; n = 4 for each group. NS, not significant, *p < 0.05 (one-way ANOVA with Tukey’s multiple comparisons test). **(B, D, F, H)** IL-33 expression was analyzed by western blotting with an anti-IL-33 antibody. The data are representative of experiments repeated three times with similar results.

To further reveal the involvement of IL-37 in the downregulation of IL-33 induced by tapinarof and GFF treatment, we knocked down IL-37 using the transfection of siRNA against AHR and applied treatment with either tapinarof (500 nM) or GFF (5%) for 48 h. qRT-PCR and western blotting analysis showed that tapinarof and GFF treatment decreased the protein levels of IL-33, which was partially reversed by the knockdown of IL-37 (**Figures 4A–D**). Furthermore, we examined whether AHR was mechanistically involved in these changes. Knockdown of AHR expression alone upregulated IL-33 in NHEKs, as previously reported (24) (**Figures 4E–H**). Similarly, knockdown of AHR partially canceled the inhibitory effects of tapinarof and GFF treatment on the protein levels of IL-33 in NHEKs (**Figures 4F, H**). To confirm this result, we utilized CH223191, a specific AHR antagonist. We treated NHEKs with either tapinarof (500 nM) or GFF (5%) in the presence or absence of CH223191 (5 μM) for 48 h. Western blotting analysis showed that CH223191 reversed the downregulation of the protein levels of IL-33 induced by tapinarof and GFF (**Supplementary Figure S5**). These results suggest that tapinarof and GFF treatment downregulates IL-33 *via* the AHR/IL-37 axis in NHEKs.

## Discussion

We have revealed the mechanism of upregulating IL-37 expression *via* AHR. Since IL-37 is an anti-inflammatory cytokine, the decrease of IL-37 expression with age reportedly is related to inflammaging, an immune senescence and chronic inflammation (25). Therefore, the strategy to control IL-37 expression using AHR modulators such as tapinarof and GFF should be beneficial for the treatment of aging as well as inflammatory skin diseases including AD and psoriasis. Our previous studies showed that nuclear factor E2-related factor 2 (NRF2), a master switch that induces antioxidant enzymes, is required for the anti-inflammatory effects of AHR activation in human keratinocytes (26, 27). Since the dysfunction of NRF2 has been reported to potentiate skin inflammation (28), activators of the AHR-NRF2 axis should be candidate therapeutic agents for inflammatory skin diseases such as AD and psoriasis (14). Specifically, tapinarof is a potential new topical treatment for AD and psoriasis as a therapeutic AHR-modulating agent (29). It has been reported that tapinarof exerts anti-inflammatory effects by activating NRF2 *via* AHR in NHEKs (30). Our results further revealed that the upregulation of IL-37 in addition to NRF2 activation is involved in tapinarof-mediated anti-inflammatory effects on the skin. Since the relationship between the regulation of IL-37 and NRF2 has not been elucidated, further studies are needed to reveal the precise mechanism involved. GFF is a natural product-derived cosmetic agent also shown to exert antioxidant effects *via* the AHR-NRF2 axis in human keratinocytes (19). Furthermore, GFF reduces aging-associated CDKN2A/p16INK4A by activating NRF2 (31). In a recent clinical study, GFF was reported to improve pore area, skin irritation, and redness when used twice daily on the face (32). Therefore, our results that GFF upregulates IL-37 in addition to NRF2 activation may provide evidence of its broad range of skin-improving effects.

PG102, a plant-derived substance extracted from *Actinidia arguta*, has also been reported to increase IL-37 expression in human keratinocytes (23). The *A. arguta* extract contains many polyphenols, with quercetin derivatives and kaempferol derivatives having been identified as the main components (33, 34). Since quercetin and kaempferol have been shown to act on AHR (35, 36), it is possible that PG102 also increases the expression of IL-37 *via* AHR. We observed the intracellular expression of IL-37 without its extracellular secretion in keratinocytes as has been reported in PG102. Therefore, it is likely that the extracellular secretion of IL-37 requires the activation of other signaling pathways, but further studies are needed on this issue. In addition, UV irradiation is effective in the treatment of AD, and notably UV irradiation increases the expression of IL-37 in the skin (37). UV irradiation onto the skin causes a conformational change of intracellular tryptophan to FICZ (38), an endogenous AHR ligand, and our previous studies confirmed that FICZ exerts a therapeutic effect on a murine AD model (39). Therefore, it is possible that the increase in IL-37 expression by UV irradiation is mediated by an AHR-dependent mechanism.

**Figure 5:**
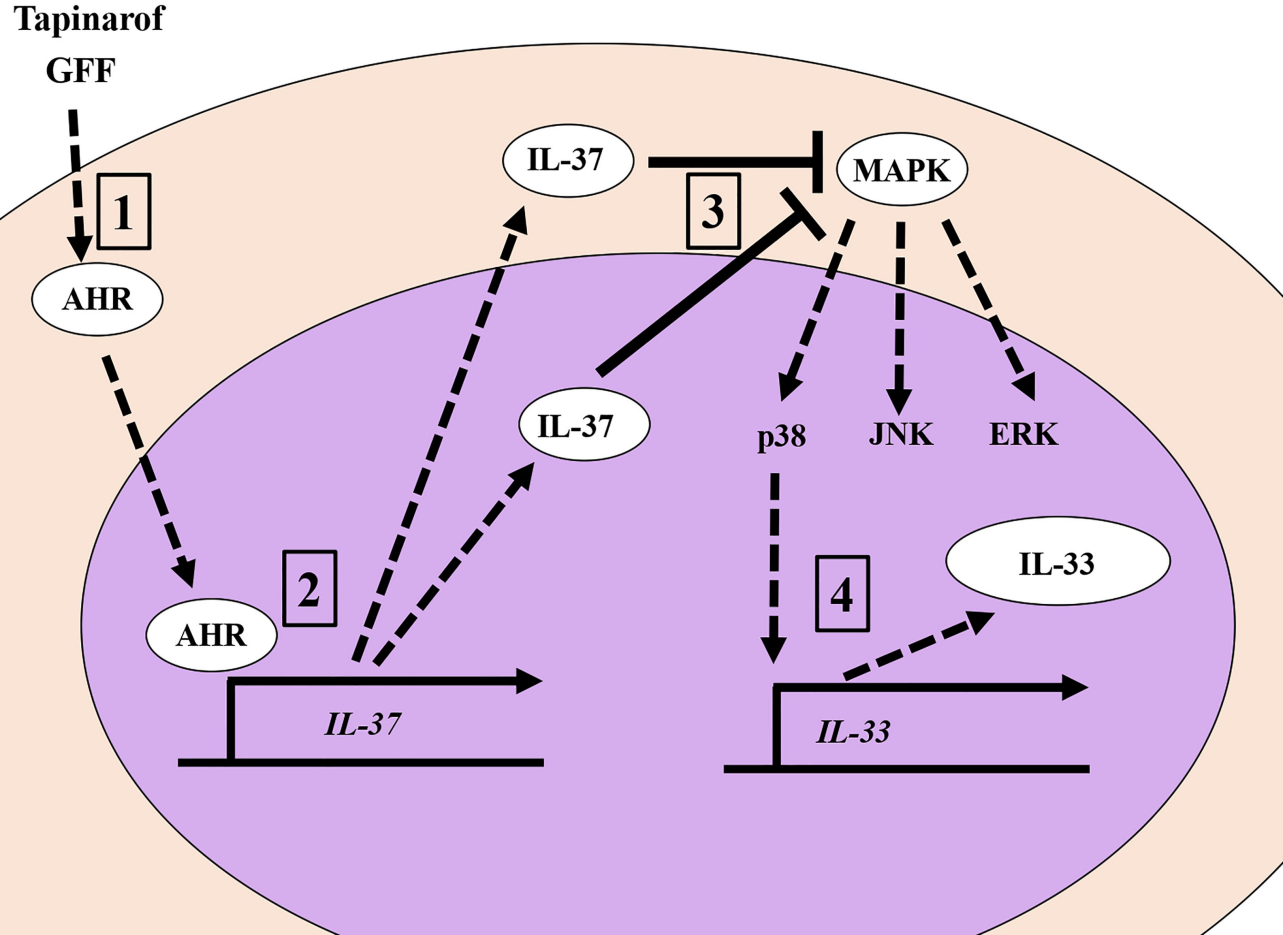
The AHR/IL-37 axis downregulates IL-33 in NHEKs. 1. Tapinarof and GFF act on AHR. 2. Translocation of AHR upregulates IL-37 ranscriptionally. 3. IL-37 negatively regulates the phosphorylation of p38 MAPKs. 4. Inhibition of p38 phosphorylation results in the downregulation of IL-33.

In this study, we demonstrated for the first time that IL-37 suppressively regulates IL-33 in human keratinocytes. Keratinocyte-derived IL-33 is deeply involved in the pathogenesis of AD since it prompts a type II immune response by activating innate lymphoid cell 2 and directly stimulating nerves, resulting in pruritus (40, 41). It has also been shown that IL-33 released from keratinocytes enhanced the transcription of genes encoding psoriasis-related cytokines, chemokines, and inflammatory molecules in keratinocytes in an autocrine manner (42, 43), suggesting that IL-33 is also a potential therapeutic target for psoriasis. IL-37 has been reported to exert its anti-inflammatory effects through an intracellular mechanism that reduces the activation of pro-inflammatory signaling mediators including p38, ERK, and STAT1 (44). Considering that IL-33 expression is partially regulated *via* ERK, p38 (45), and STAT1 (46), it is possible that IL-37 downregulates IL-33 by suppressing potential MAPK and STAT1 activation in human keratinocytes. Indeed, the knockdown of IL-37 expression by siRNA transfection was shown to induce the phosphorylation of p38 and JNK. Our previous studies showed that the phosphorylation of ERK and p38 is involved in the expression of IL-33 in keratinocytes, while the phosphorylation of JNK has no significant effect (24). These results suggest that the suppression of p38 phosphorylation by IL-37 acts in the direction of suppressing IL-33 expression (**Figure 5**).

Accordingly, we have shown that tapinarof and GFF, which act on AHR, increased the expression of IL-37, which in turn suppressed the expression of IL-33, a crucial cytokine in the development of AD and psoriasis. We believe that the AHR/IL-37 axis will be important for future therapeutic strategies for treating AD and psoriasis.

## Data Availability Statement

The original contributions presented in the study are publicly available. This data can be found here: https://www.ncbi.nlm.nih.gov/geo/, GSE181089.

## Author Contributions

GT designed the experiments. GT, MF, and TN wrote the manuscript. GT, AH-H, TM-T, AT-Y, and MT performed the experiments. GT, XY, and MF analyzed the results. All authors contributed to the interpretation of the research. All authors contributed to the article and approved the submitted version.

## Funding

This work was partly supported by grants from the Ministry of Health, Labor, and Welfare, Japan (R3-Shokuhin-Shitei-005), and JSPS KAKENHI (grant number 20K08653).

## Conflict of Interest

GT obtained a research grant from Procter & Gamble Innovation Godo Kaisha. MF is a consultant at Procter & Gamble Innovation Godo Kaisha. XY is employed by Procter & Gamble Innovation Godo Kaisha.

The remaining authors declare that the research was conducted in the absence of any commercial or financial relationships that could be construed as a potential conflict of interest.

## Publisher’s Note

All claims expressed in this article are solely those of the authors and do not necessarily represent those of their affiliated organizations, or those of the publisher, the editors and the reviewers. Any product that may be evaluated in this article, or claim that may be made by its manufacturer, is not guaranteed or endorsed by the publisher.
