# Supplementary figures and images for: Natural Compounds Tapinarof and Galactomyces Ferment Filtrate Downregulate IL-33 Expression via the AHR/IL-37 Axis in Human Keratinocytes
Source: Front Immunol. 2022 May 19;13:745997. doi: 10.3389/fimmu.2022.745997 (PMC9161696; doi:10.3389/fimmu.2022.745997)

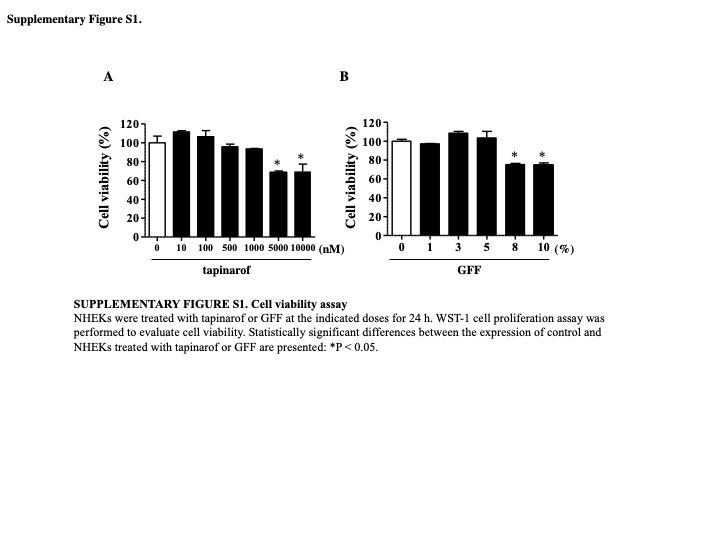

Supplement: Supplementary file 1 [file Image_1.jpeg]

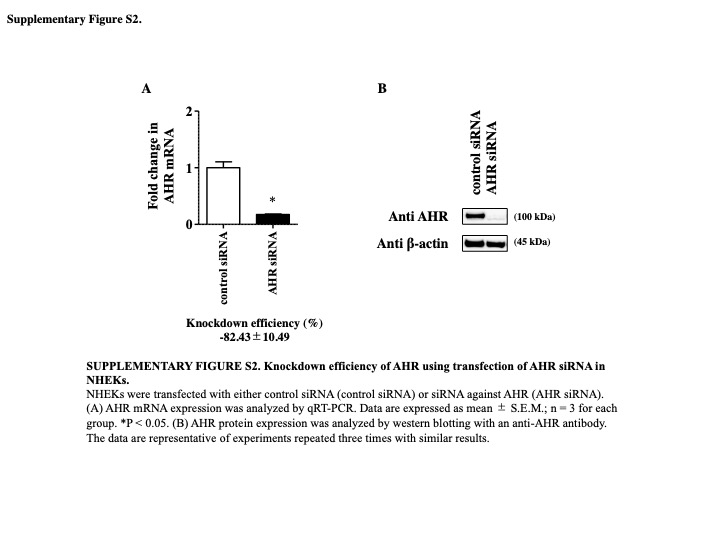

Supplement: Supplementary file 2 [file Image_2.jpeg]

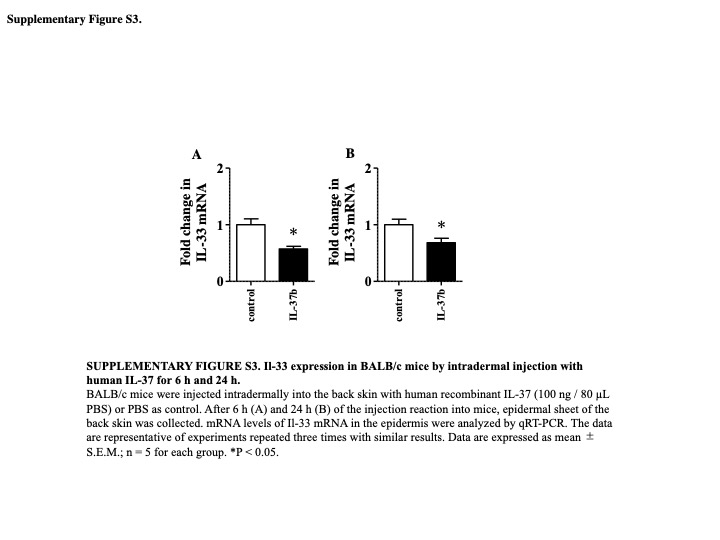

Supplement: Supplementary file 3 [file Image_3.jpeg]

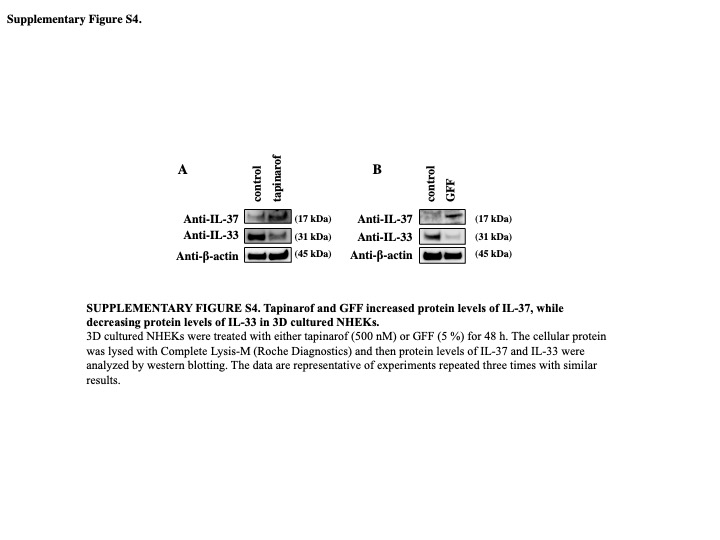

Supplement: Supplementary file 4 [file Image_4.jpeg]

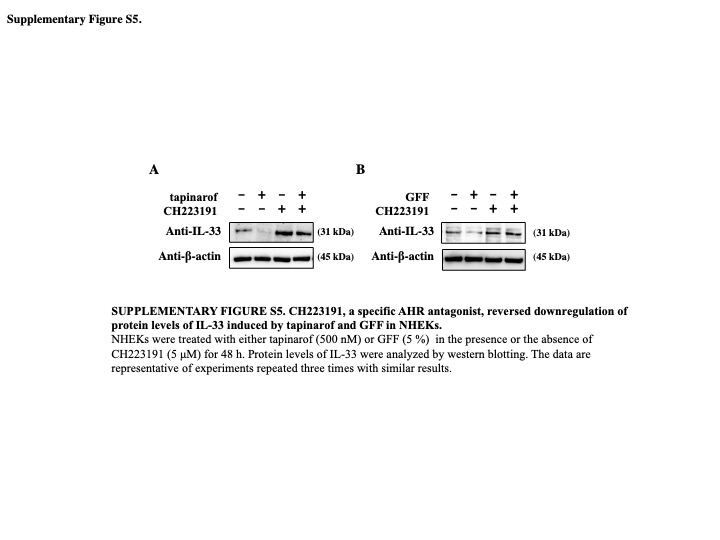

Supplement: Supplementary file 5 [file Image_5.jpeg]
