# Supplementary material for: Natural Compounds Tapinarof and Galactomyces Ferment Filtrate Downregulate IL-33 Expression via the AHR/IL-37 Axis in Human Keratinocytes
Source: Front Immunol. 2022 May 19;13:745997. doi: 10.3389/fimmu.2022.745997 (PMC9161696; doi:10.3389/fimmu.2022.745997)
Supplement: Supplementary file 6 [file Table_1.pdf]

SUPPLEMENTARY TABLE 1. Primer sequences for qRT-PCR.

Gene	Forward primer	Reverse primer
Human AHR	5′-ATCACCTACGCCAGTCGCAAG-3′	5′-AGGCTAGCCAAACGGTCCAAC-3′
Human IL33	5′-AGCCTTGTGTTTCAAGCTGGG-3′	5′-TTGTGCTTTCTACCTGTTTTCAGTG-3′
Human IL36G	5′-CAGAACCTTGTGGCAGTTCC-3′	5′-TCTGCTCTTTTAGCTGCAATGTGG-3′
Human β-actin	5′-ATTGCCGACAGGATGCAGA-3′	5′-GAGTACTTGCGCTCAGGAGGA-3′
Human IL37	Hs00367201_m1 (TaqMan™ Gene Expression Assay)	
Human YWHAZ	Hs01122445_g1 (TaqMan™ Gene Expression Assay)	
Murine IL33	5′-TGCAGGAAAGTACAGCATTCAAG-3′	5′-TTGGTCTTCTGTTGGGATCTTCTTA-3′
Murine β-actin	5′-GGCTGTATTCCCCTCCATCG-3′	5′-CCAGTTGGTAACAATGCCATGT -3′
