# Supplementary material for: Natural Compounds Tapinarof and Galactomyces Ferment Filtrate Downregulate IL-33 Expression via the AHR/IL-37 Axis in Human Keratinocytes
Source: Front Immunol. 2022 May 19;13:745997. doi: 10.3389/fimmu.2022.745997 (PMC9161696; doi:10.3389/fimmu.2022.745997)
Supplement: Supplementary file 7 [file Table_2.pdf]

SUPPLEMENTARY TABLE 2. Top 75 upregulated genes in microarray analysis.

List of the 75 genes most strongly upregulated in NHEKs with IL-37 knockdown

Gene Symbol	Ratio	P-value	Gene Description
MYLK	12.9501	0.01972	myosin light chain kinase
GCLC	8.1532	0.03745	glutamate-cysteine ligase, catalytic subunit
IL33	6.6172	0.04737	interleukin 33
AKR1C3	4.8196	0.03844	aldo-keto reductase family 1, member C3
TFRC	4.7127	0.00005	transferrin receptor
PPP2R2B	4.57	0.00605	protein phosphatase 2, regulatory subunit B, beta
ETS2	4.3672	0.03941	v-ets avian erythroblastosis virus E26 oncogene homolog 2
SAA1	4.308	0.01106	serum amyloid A1
AKR1C1	3.98	0.04965	aldo-keto reductase family 1, member C1
SAA2	3.8837	0.01195	serum amyloid A2
SOD2	3.7841	0.03126	superoxide dismutase 2, mitochondrial
PRRG4	3.6732	0.02507	proline rich Gla (G-carboxyglutamic acid) 4 (transmembrane)
SERPINB3	3.6636	0.00605	serpin peptidase inhibitor, clade B (ovalbumin), member 3
TMEM117	3.6578	0.00291	transmembrane protein 117
CCL20	3.5799	0.00139	chemokine (C-C motif) ligand 20
SATL1	3.5222	0.00825	spermidine/spermine N1-acetyl transferase-like 1
MTUS1	3.467	0.00053	microtubule-associated tumor suppressor 1
KCNK10	3.4614	0.01905	potassium channel, two pore domain subfamily K, member 10
SERPINB4	3.399	0.00146	serpin peptidase inhibitor, clade B (ovalbumin), member 4
PALMD	3.2654	0.01397	palmdelphin

**HSPB1**	**3.1603**	**0.01541**	**heat shock 27 kDa protein 1**
**FABP5**	**3.0902**	**0.04746**	**fatty acid binding protein 5 (psoriasis-associated)**
**FBXO33**	**3.0515**	**0.0152**	**F-box protein 33**
**SESN1**	**2.972**	**0.00206**	**sestrin 1**
**GBP1**	**2.853**	**0.02152**	**guanylate binding protein 1, interferon-inducible**
**DSC3**	**2.8437**	**0.03378**	**desmocollin 3**
**BTG2**	**2.8178**	**0.04646**	**BTG family, member 2**
**TNFSF10**	**2.7982**	**0.04818**	**tumor necrosis factor (ligand) superfamily, member 10**
**VWA9**	**2.7819**	**0.00042**	**von Willebrand factor A domain-containing 9**
**STON2**	**2.7817**	**0.00046**	**stonin 2**
**BMP2K**	**2.714**	**0.00557**	**BMP2 inducible kinase**
**NTN4**	**2.6493**	**0.01716**	**netrin 4**
**UBE2D1**	**2.5971**	**0.00062**	**ubiquitin-conjugating enzyme E2D 1**
**CARS2**	**2.5807**	**0.00109**	**cysteinyl-tRNA synthetase 2, mitochondrial (putative)**
**TMTC1**	**2.5604**	**0.0022**	**transmembrane and tetratricopeptide repeat containing 1**
**CA2**	**2.5135**	**0.03113**	**carbonic anhydrase II**
**SKAP2**	**2.5021**	**0.01466**	**src kinase-associated phosphoprotein 2**
**YBX3**	**2.4731**	**0.01318**	**Y box binding protein 3**
**PRKAB2**	**2.4618**	**0.01406**	**protein kinase, AMP-activated, beta 2 non-catalytic subunit**
**NAB1**	**2.4426**	**0.01258**	**NGFI-A binding protein 1**
**NFKBIA**	**2.4283**	**0.04856**	**nuclear factor of kappa light polypeptide gene enhancer in B cells inhibitor, alpha**
**KANK4**	**2.4235**	**0.03607**	**KN motif and ankyrin repeat domains 4**
**CYP1A1**	**2.3907**	**0.04358**	**cytochrome P450, family 1, subfamily A, polypeptide 1**

**EFNA5**	**2.3456**	**0.03414**	**ephrin-A5**
**IL36G**	**2.3168**	**0.03462**	**interleukin 36, gamma**
**PAMR1**	**2.2968**	**0.03713**	**peptidase domain-containing associated with muscle regeneration 1**
**FDXR**	**2.2353**	**0.0432**	**ferredoxin reductase**
**RPS6KA5**	**2.2282**	**0.01667**	**ribosomal protein S6 kinase, 90 kDa, polypeptide 5**
**COL1A1**	**2.2037**	**0.00435**	**Jeck2013 ANTISENSE, coding, INTERNAL, intronic best transcript NM_000088**
**ACER2**	**2.1992**	**0.00876**	**alkaline ceramidase 2**
**ST13**	**2.1904**	**0.0012**	**suppression of tumorigenicity 13 (colon carcinoma) (Hsp70-interacting protein)**
**VSNL1**	**2.1739**	**0.02962**	**visinin-like 1**
**SEPW1**	**2.1694**	**0.02028**	**selenoprotein W, 1**
**VPS26B**	**2.1529**	**0.00161**	**VPS26 retromer complex component B**
**TCP10L2**	**2.1453**	**0.01372**	**t-complex 10-like 2**
**SATB1**	**2.1438**	**0.03775**	**SATB homeobox 1**
**SPRYD4**	**2.1386**	**0.02228**	**SPRY domain-containing 4**
**KCNAB2**	**2.1055**	**0.00978**	**potassium channel, voltage-gated subfamily A regulatory beta subunit 2**
**RNF128**	**2.0956**	**0.03017**	**ring finger protein 128, E3 ubiquitin protein ligase**
**XPR1**	**2.0912**	**0.03464**	**xenotropic and polytropic retrovirus receptor 1**
**SLC19A2**	**2.0877**	**0.01439**	**solute carrier family 19 (thiamine transporter), member 2**
**AHR**	**2.0775**	**0.00639**	**aryl hydrocarbon receptor**
**FAM8A1**	**2.0758**	**0.03181**	**family with sequence similarity 8, member A1**
**CA12**	**2.0725**	**0.01321**	**carbonic anhydrase XII**
**SSBP2**	**2.0623**	**0.03544**	**single-stranded DNA binding protein 2**

**TSHZ2**	**2.0623**	**0.0128**	**teashirt zinc finger homeobox 2**
**WIPI1**	**2.0534**	**0.01921**	**WD repeat domain, phosphoinositide-interacting 1**
**OR3A3**	**2.0524**	**0.03036**	**olfactory receptor, family 3, subfamily A, member 3**
**PIK3IP1**	**2.0496**	**0.01751**	**phosphoinositide-3-kinase-interacting protein 1**
**STOM**	**2.0462**	**0.00524**	**stomatin**
**OR5AL1**	**2.0341**	**0.01094**	**olfactory receptor, family 5, subfamily AL, member 1 (gene/pseudogene)**
**ZNF608**	**2.0316**	**0.00792**	**zinc finger protein 608**
**GOPC**	**2.0294**	**0.01245**	**Golgi-associated PDZ and coiled-coil motif-containing**
**IFNGR1**	**2.0171**	**0.02831**	**interferon gamma receptor 1**
**SETD7**	**2.0003**	**0.01215**	**SET domain-containing (lysine methyltransferase) 7**
