# Supplementary material for: Natural Compounds Tapinarof and Galactomyces Ferment Filtrate Downregulate IL-33 Expression via the AHR/IL-37 Axis in Human Keratinocytes
Source: Front Immunol. 2022 May 19;13:745997. doi: 10.3389/fimmu.2022.745997 (PMC9161696; doi:10.3389/fimmu.2022.745997)
Supplement: Supplementary file 8 [file Table_3.pdf]

SUPPLEMENTARY TABLE 3. Top 100 downregulated genes in microarray analysis.

List of the 100 genes most strongly downregulated in NHEKs with IL-37 knockdown

Gene Symbol	Ratio	P-value	Gene Description
BMP4	0.0893	0.00568	bone morphogenetic protein 4
CTGF	0.1246	0.0068	connective tissue growth factor
GNG5	0.1252	0.00002	guanine nucleotide binding protein (G protein), gamma 5
DUSP4	0.1407	0.00325	dual-specificity phosphatase 4
CMTM7	0.1425	0.00516	CKLF-like MARVEL transmembrane domain-containing 7
PPIF	0.149	0.00123	peptidylprolyl isomerase F
TMEM200A	0.1586	0.04305	transmembrane protein 200A
SPCS3	0.1612	0.00232	signal peptidase complex subunit 3
HN1L	0.1655	0.00133	hematological and neurological expressed 1-like
EPN3	0.184	0.04466	epsin 3
CLDN4	0.1868	0.01336	claudin 4
FAM214B	0.1911	0.00134	family with sequence similarity 214, member B
DPYSL2	0.1942	0.03111	dihydropyrimidinase-like 2
ANAPC13	0.2014	0.00002	anaphase-promoting complex subunit 13
SPRY4	0.2059	0.03886	sprouty RTK signaling antagonist 4
HYOU1	0.2089	0.00186	hypoxia up-regulated 1
SMURF2	0.2148	0.01653	SMAD-specific E3 ubiquitin protein ligase 2
DKK1	0.2159	0.03508	Dickkopf WNT signaling pathway inhibitor 1
BCL2L1	0.2163	0.01833	BCL2-like 1
CDK6	0.2167	0.01011	cyclin-dependent kinase 6
TGM2	0.2201	0.0363	transglutaminase 2
RCAN3	0.2218	0.00637	RCAN family member 3

**PLAUR**	**0.2239**	**0.00126**	**plasminogen activator, urokinase receptor**
**DNAJC8**	**0.2243**	**0.00123**	**DnaJ (Hsp40) homolog, subfamily C, member 8**
**CYB561**	**0.2249**	**0.00125**	**cytochrome b561**
**MTMR1**	**0.2331**	**0.00553**	**myotubularin related protein 1**
**PLSCR1**	**0.2371**	**0.00107**	**phospholipid scramblase 1**
**TGFBR2**	**0.2373**	**0.01726**	**transforming growth factor beta receptor II**
**HAS2**	**0.2397**	**0.04651**	**hyaluronan synthase 2**
**VCL**	**0.2449**	**0.01027**	**vinculin**
**C9orf64**	**0.2463**	**0.01556**	**chromosome 9 open reading frame 64**
**SSRP1**	**0.2475**	**0.00508**	**structure-specific recognition protein 1**
**TMEM179B**	**0.2503**	**0.00016**	**transmembrane protein 179B**
**TMEM41B**	**0.2535**	**0.00177**	**transmembrane protein 41B**
**XPNPEP3**	**0.2561**	**0.02263**	**X-prolyl aminopeptidase 3, mitochondrial**
**CAMK2N1**	**0.2565**	**0.00272**	**calcium/calmodulin-dependent protein kinase II inhibitor 1**
**FAM83A**	**0.269**	**0.03382**	**family with sequence similarity 83, member A**
**SLC43A3**	**0.269**	**0.03293**	**solute carrier family 43, member 3**
**MAOA**	**0.2741**	**0.00524**	**monoamine oxidase A**
**DGCR2**	**0.2772**	**0.01223**	**DiGeorge syndrome critical region gene 2**
**RAB3B**	**0.2781**	**0.01181**	**RAB3B, member RAS oncogene family**
**TGFA**	**0.2818**	**0.00399**	**transforming growth factor alpha**
**ARHGAP18**	**0.2826**	**0.00051**	**Rho GTPase activating protein 18**
**PLIN3**	**0.2841**	**0.00157**	**perilipin 3**
**SERPINE1**	**0.2843**	**0.03609**	**serpin peptidase inhibitor, clade E (nexin, plasminogen activator inhibitor type 1), member 1**
**DUSP5**	**0.2901**	**0.00097**	**dual-specificity phosphatase 5**

**CASP2**	**0.2918**	**0.00531**	**caspase 2**
**FAM162A**	**0.2929**	**0.00029**	**family with sequence similarity 162, member A**
**TSN**	**0.2961**	**0.00088**	**translin**
**PGK1**	**0.2967**	**0.00411**	**phosphoglycerate kinase 1**
**CITED4**	**0.2976**	**0.01114**	**Cbp/p300-interacting transactivator, with Glu/Asp-rich carboxy-terminal domain, 4**
**TMEM64**	**0.2987**	**0.00783**	**transmembrane protein 64**
**CXCL1**	**0.3011**	**0.02125**	**chemokine (C-X-C motif) ligand 1 (melanoma growth-stimulating activity, alpha)**
**RBBP5**	**0.3021**	**0.01326**	**retinoblastoma binding protein 5**
**LTBP4**	**0.3057**	**0.01762**	**latent transforming growth factor beta binding protein 4**
**B3GNT2**	**0.3068**	**0.03771**	**UDP-GlcNAc:betaGal beta-1,3-N-acetylglucosaminyltransferase 2**
**GRB2**	**0.3081**	**0.00456**	**growth factor receptor bound protein 2**
**SLC16A3**	**0.3094**	**0.00229**	**solute carrier family 16 (monocarboxylate transporter), member 3**
**WNT7A**	**0.3097**	**0.01601**	**wingless-type MMTV integration site family, member 7A**
**MCFD2**	**0.3119**	**0.00343**	**multiple coagulation factor deficiency 2**
**AXL**	**0.3121**	**0.01695**	**AXL receptor tyrosine kinase**
**TSPYL1**	**0.3135**	**0.00285**	**TSPY-like 1**
**PSMB3**	**0.315**	**0.00622**	**proteasome subunit beta 3**
**NRP1**	**0.3198**	**0.03248**	**neuropilin 1**
**MMD**	**0.3209**	**0.00698**	**monocyte to macrophage differentiation-associated**
**ANO6**	**0.329**	**0.00498**	**anoctamin 6**
**TNFSF15**	**0.3291**	**0.03468**	**tumor necrosis factor (ligand) superfamily, member 15**
**SLC16A1**	**0.3305**	**0.00022**	**solute carrier family 16 (monocarboxylate transporter), member 1**
**EPHA2**	**0.332**	**0.00779**	**EPH receptor A2**
**NMT2**	**0.3352**	**0.00058**	**N-myristoyltransferase 2**

**TGFBR1**	**0.338**	**0.03946**	**transforming growth factor, beta receptor 1**
**BLCAP**	**0.3387**	**0.00668**	**bladder cancer-associated protein**
**NET1**	**0.3403**	**0.00008**	**neuroepithelial cell transforming 1**
**PLAU**	**0.3421**	**0.00057**	**plasminogen activator, urokinase**
**ETV5**	**0.3431**	**0.01996**	**ets variant 5**
**PAAF1**	**0.3433**	**0.01127**	**proteasomal ATPase-associated factor 1**
**GABARAPL2**	**0.3435**	**0.00158**	**GABA(A) receptor-associated protein like 2**
**SEC61G**	**0.3442**	**0.00105**	**Sec61 translocon gamma subunit**
**CDCA5**	**0.3447**	**0.04401**	**cell division cycle-associated 5**
**KCTD12**	**0.3465**	**0.00862**	**potassium channel tetramerization domain-containing 12**
**EMP1**	**0.3467**	**0.03866**	**epithelial membrane protein 1**
**ANKRD36**	**0.3484**	**0.043**	**ankyrin repeat domain 36**
**MARVELD1**	**0.3509**	**0.01942**	**MARVEL domain-containing 1**
**HGSNAT**	**0.3524**	**0.00524**	**heparan-alpha-glucosaminide N-acetyltransferase**
**RAB8B**	**0.3529**	**0.01614**	**RAB8B, member RAS oncogene family**
**ATP6V0D1**	**0.3557**	**0.00059**	**ATPase, H^+-transporting, lysosomal 38 kDa, V0 subunit d1**
**ITGA5**	**0.3558**	**0.04765**	**integrin alpha 5**
**SEMA7A**	**0.356**	**0.01731**	**semaphorin 7A, GPI membrane anchor (John Milton Hagen blood group)**
**CDC34**	**0.3579**	**0.01525**	**cell division cycle 34**
**RUBCN**	**0.3582**	**0.031**	**RUN domain and cysteine-rich domain containing, Beclin 1-interacting protein**
**SFRP1**	**0.3586**	**0.02001**	**secreted frizzled-related protein 1**
**C3orf14**	**0.3596**	**0.03504**	**chromosome 3 open reading frame 14**
**TCEB1**	**0.3607**	**0.00202**	**transcription elongation factor B (SIII), polypeptide 1 (15 kDa, elongin C)**
**SHISA5**	**0.364**	**0.0015**	**Shisa family member 5**

**LASP1**	**0.3657**	**0.02078**	**LIM and SH3 protein 1**
**RHOBTB2**	**0.3678**	**0.00578**	**Rho-related BTB domain-containing 2**
**DUT**	**0.368**	**0.04197**	**deoxyuridine triphosphatase**
**ATL3**	**0.3696**	**0.01695**	**atlastin GTPase 3**
**GPD2**	**0.3696**	**0.03897**	**glycerol-3-phosphate dehydrogenase 2**
**ANTXR2**	**0.3707**	**0.01128**	**anthrax toxin receptor 2**
